# P-1769. Malaria on the Move: Rising cases in Brooklyn, New York

**DOI:** 10.1093/ofid/ofaf695.1939

**Published:** 2026-01-11

**Authors:** Saira P Iqbal, Madalasa Pokhrel, Rinita reddi, Jessica Chung, Tina Zheng, Monica Ghitan, Edward Chapnick, Yu Shia Lin

**Affiliations:** Maimonides Health, Brooklyn, NY; Maimonides Health, Brooklyn, NY; Maimonides Health, Brooklyn, NY; Maimonides Health, Brooklyn, NY; Maimonides Health, Brooklyn, NY; Maimonides Medical Center, New York, New York; Maimonides Medical Center, New York, New York; Maimonides Medical Center, New York, New York

## Abstract

**Background:**

Malaria is an endemic disease in 85 countries, with 263 million cases reported worldwide in 2023. This represents a 4% increase from 2022. A similar trend was observed in our hospital with a significant increase in malaria cases presenting over the past 2 years, especially in migrants. This study aims to analyze this trend and describe patient and microbiologic characteristics.

**Figure:**
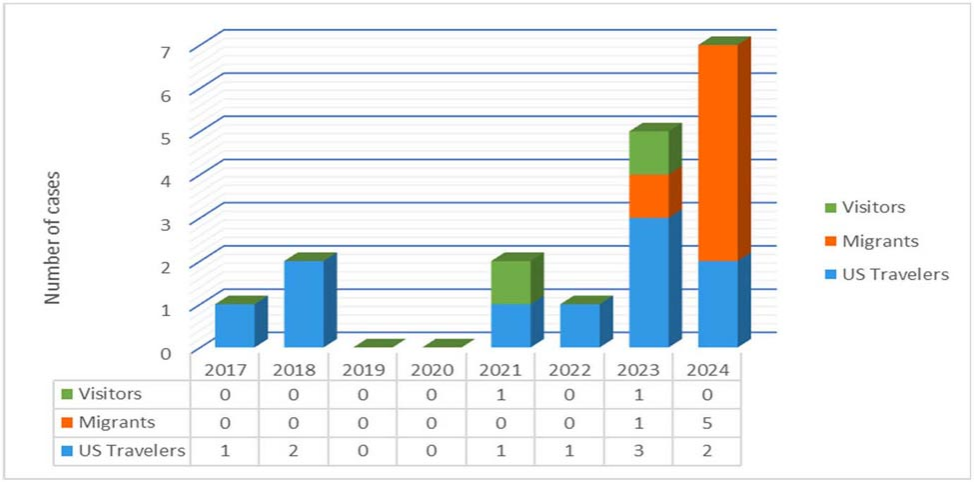
Rising trends in Malaria cases from 2017 to 2024

**Figure:**
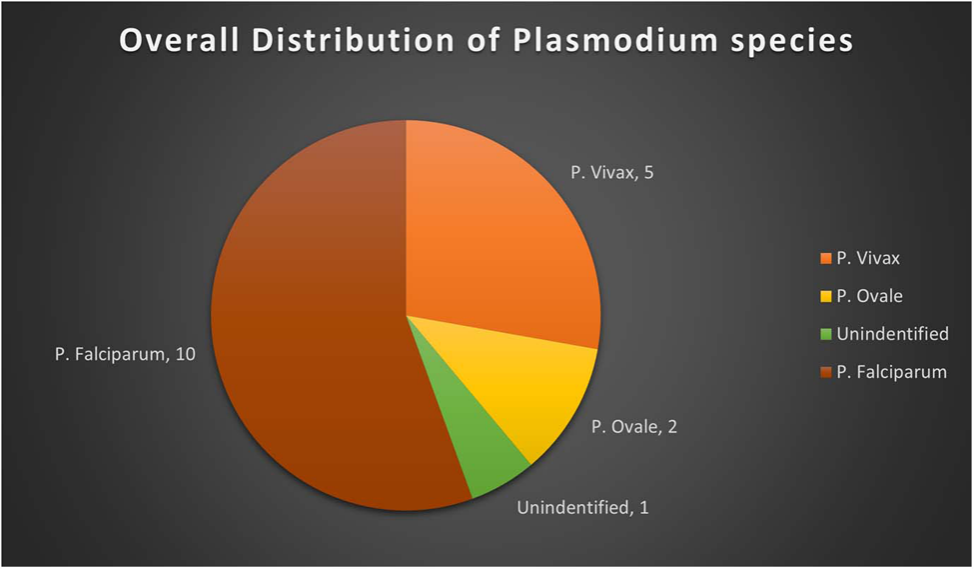
Overall distribution of Plasmodium species

**Methods:**

We conducted a retrospective chart review of patients admitted with confirmed malaria between 2017 and 2024. Patients' travel history, country of origin, clinical course, microbiology results and treatment outcomes were recorded. Malaria diagnosis was confirmed using one or more of the following tests:

- Peripheral blood smear
- Rapid antigen test ((BinaxNOW™ Malaria, Abbott, Chicago, IL)
- *Plasmodium* species polymerase chain reaction (PCR) test

**Figure:**
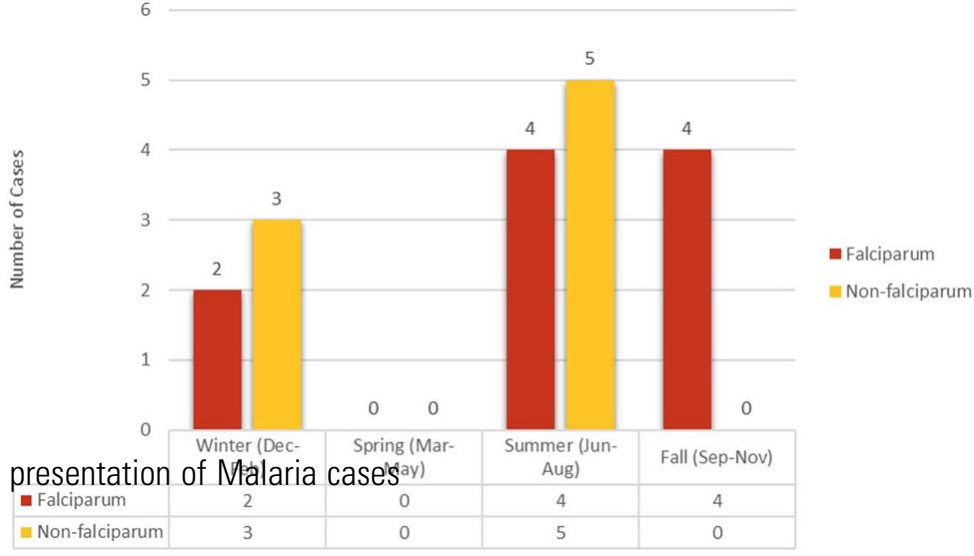
Seasonal presentation of Malaria cases

**Figure:**
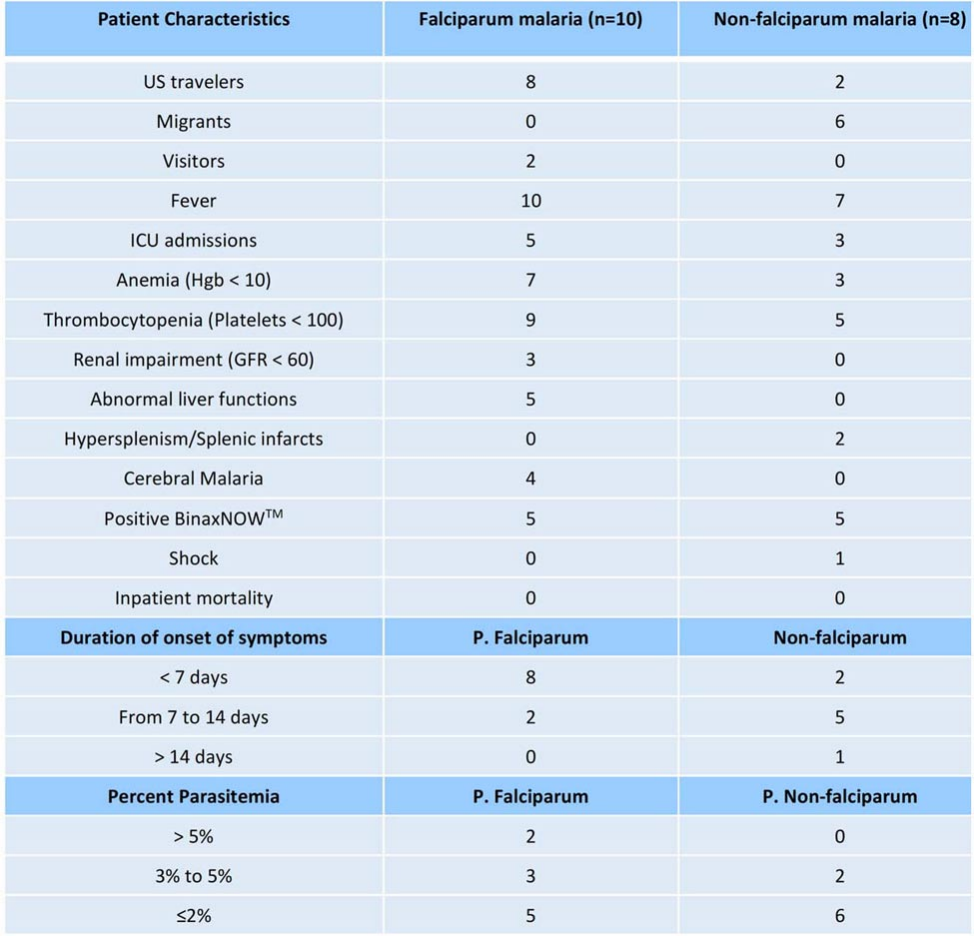
Comparison between Falciparum and Non-falciparum groups

**Results:**

Total of 18 patients were included in the study. Figure 1 shows the number of cases admitted per year and trends between US travelers, visitors and migrants. This study also reviewed the overall prevalence of *Plasmodium* species (Figure 2) and its association with seasonal trends (Figure 3). Patients were categorized into two groups:

• Patients with Falciparum malaria

• Patients with non-falciparum malaria

Clinical characteristics between two groups have been summarized in Table A.

In the non-falciparum group, 6 patients had migrated through the Darién rainforest from Colombia to Panama. Initial parasitemia was higher in *P. falciparum* group compared to *non-falciparum group.* Most *P. falciparum* cases presented within 7 days of travel, while most non-falciparum cases presented between 7-14 days. Eight patients had severe malaria; 5 were infected with *P. falciparum* and 3 with *P. vivax*. They were treated with intravenous artesunate, followed by oral artemether/lumefantrine. All patients recovered uneventfully.

**Conclusion:**

Malaria cases in USA have increased over last few years, especially among returning U.S travelers and migrants. *P. falciparum* is predominant in West and Central Africa, whereas *P. vivax* and *P. ovale* is endemic in South America. This rise may be attributable to migrants crossing the Darién rainforest between Colombia and Panama. A detailed travel history may help anticipate the *Plasmodium* species for timely diagnosis and prompt management.

**Disclosures:**

All Authors: No reported disclosures

